# The serum free triiodothyronine to free thyroxine ratio as a potential prognostic biomarker of chronic kidney disease in patients with glomerular crescents: A retrospective study

**DOI:** 10.3389/fendo.2022.977355

**Published:** 2022-09-29

**Authors:** Liwen Zhang, Yuxiao Wu, Yuxin Nie, Wenlv Lv, Yang Li, Bowen Zhu, Shi Jin, Ziyan Shen, Fang Li, Hong Liu, Yi Fang, Xiaoqiang Ding

**Affiliations:** ^1^ Department of Nephrology, Zhongshan Hospital, Fudan University, Shanghai, China; ^2^ Shanghai Institute of Kidney and Dialysis, Zhongshan Hospital, Fudan University, Shanghai, China; ^3^ Shanghai Medical Center of Kidney, Zhongshan Hospital, Fudan University, Shanghai, China; ^4^ Shanghai Key Laboratory of Kidney and Blood Purification, Zhongshan Hospital, Fudan University, Shanghai, China; ^5^ Department of Internal Medicine, Zhongshan Hospital, Fudan University, Shanghai, China

**Keywords:** glomerular crescents, chronic kidney disease, FT3/FT4 ratio, prognosis, thyroid function

## Abstract

**Background:**

Crescent formation indicates severe glomerular pathology, and hypothyroidism usually predicts poor prognosis for severe diseases. However, the relationship between thyroid function and the progression of chronic kidney disease (CKD) is unclear. This study analysed the prognostic predictive value of the serum free triiodothyronine (FT3) to free thyroxine (FT4) ratio and its correlation with renal function in patients with CKD with crescent formation.

**Methods:**

This single-centre study included 162 CKD patients with glomerular crescents confirmed by renal pathology between March 2012 and December 2014. According to the first tertile (0.284) of FT3/FT4 ratio, the patients were divided into high and low FT3/FT4 ratio groups. Kaplan-Meier and Cox regression analyses were performed to evaluate the prognostic value of the FT3/FT4 ratio.

**Results:**

The age, haemoglobin, eGFR, urinary albumin-to-creatinine ratio, cardiac troponin T, N-terminal brain natriuretic peptide precursor, FT3, FT4, percentage of total crescents in non-globally sclerotic glomeruli, prevalences of hypertension, moderate to severe renal tubulopathy and crescentic nephritis, and proportion of patients receiving glucocorticoids and immunosuppressants were significantly different between high and low FT3/FT4 ratio groups (P < 0.05). Multivariate Cox regression analysis showed that when compared with patients with a high FT3/FT4 ratio (>0.284), those with intermediate and low FT3/FT4 ratios (≤0.284) had an increased risk of the long-term composite endpoint (P < 0.05 for various adjustment models).

**Conclusions:**

A low FT3/FT4 ratio is associated with increased mortality and worse outcome risk in CKD patients with crescent pathology.

## Introduction

Glomerular crescent formation is a hallmark of severe glomerular injury and can present in patients with primary and secondary glomerulonephritis. The presence of extensive glomerular crescents (usually greater than 50%) is the main pathological feature of rapidly progressive glomerulonephritis. Several studies (1, 2) have suggested that crescent formation is an independent predictor of a poor renal prognosis. Therefore, crescent was added to the MEST score in the Oxford Classification of IgA nephropathy that was updated in 2017 (3). However, there is insufficient evidence to base treatment decisions on the presence or number of crescents in a kidney biopsy (4). Therefore, we need to explore new predictors to assess the risk of outcomes in chronic kidney disease (CKD) patients with crescent formation and identify target populations that require early aggressive intervention.

‘Non-thyroidal illness syndrome’ (NTIS) is a syndrome of abnormal thyroid hormone metabolism that is general in critically ill patients and has been also reported in patients with CKD. Thyroxine (T4) is the main secreted and transported form of thyroid hormone. Its free form can be transported into cells to be de-iodinated and converted into free triiodothyronine (FT3), which regulates energy metabolism and protein synthesis and stimulates tissue growth, maturation, and differentiation. Impaired FT4-FT3 conversion is a major characteristic of NTIS, and the decreased FT3/FT4 ratio is therefore its biomarker. NITS is associated with decreased renal function in non-dialysis CKD patients (5, 6) and also predicts adverse outcomes in renal replacement therapy, such as kidney graft failure (7) and cardiovascular events in haemodialysis (HD) patients (8). However, a retrospective study of 317 patients with nephrotic syndrome failed to demonstrate the association between renal pathological type and NITS, marked as low FT3 levels or hypothyroidism (9).

Therefore, to clarify the association between the degree of T4-T3 conversion and the progression of CKD with crescents, the severe glomerular pathological changes suggesting high disease activity and rapidly progression, our study assessed the association of the FT3/FT4 ratio with a composite endpoint in a retrospective cohort of CKD patients with crescent formation in our centre.

## Materials and methods

### Study participants

**Figure 1:**
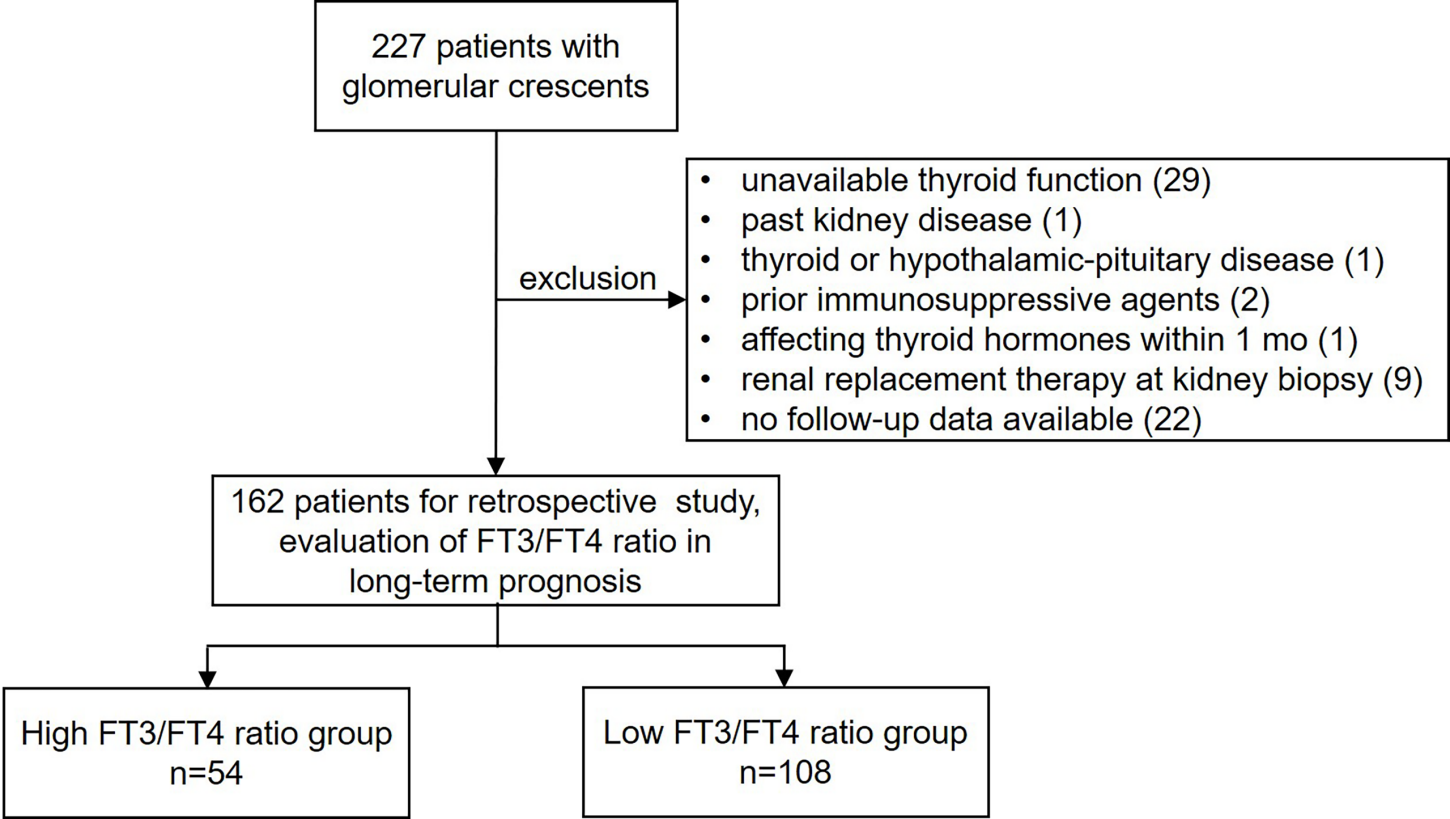
Overall design of the study.

We analysed 227 consecutive patients who underwent kidney biopsy and showed glomerular crescent pathology at the Department of Nephrology, Zhongshan Hospital, Fudan University, between March 2012 and December 2014. Demographic variables (age and sex), clinical variables, laboratory tests results, kidney pathology results, and pharmacological treatment were obtained from electronic medical records. The inclusion criteria were as follows: (i) age ≥18 years at biopsy and (ii) renal biopsy-proven crescent formation (including cellular/fibrocellular/fibrous crescents). The exclusion criteria were as follows: (i) unavailable thyroid functional test results within 1 week before kidney biopsy; (ii) past history of kidney disease; (iii) past history of thyroid disease or hypothalamic-pituitary disease; (iv) immunosuppressive therapy before kidney biopsy; (v) administration of medication that may affect thyroid hormone secretion and metabolism within 1 month of the thyroid function test; (vi) renal replacement therapy (RRT) at kidney biopsy; and (vii) no follow-up data available. This study was approved by the ethics review committee of Zhongshan Hospital, Fudan University. The overall design of this study is shown in **Figure 1**.

### Measurements and definitions

Hypertension was defined as systolic blood pressure ≥140 mmHg and/or diastolic blood pressure ≥90 mmHg at repeated examinations on different days in the hospital, self-reported history of hypertension, or use of antihypertensive medications (10).

The estimated glomerular filtration rate (eGFR) was calculated using the 2009 CKD-EPI creatinine formula (11).

Kidney biopsy reports were based on independent grading by two histopathologists. Glomerular crescents were defined as hyperplastic lesions involving >10% of the circumference of the Bowman’s capsule, including cellular, fibrocellular, and fibrous crescents. A ‘cellular crescent’ was defined as extracapillary hypercellularity of >2 cell layers composed of >75% cells with or without fibrin and <25% fibrous matrix. A composition of 25–75% cells with or without fibrin and the remaining fibrous matrix was defined as a ‘fibrocellular crescent’, and a composition of >75% fibrous matrix and <25% cells with or without fibrin was defined as a ‘fibrous crescent’ (12). The extent of interstitial fibrosis and tubular atrophy (IFTA) was graded on the estimated percentage of IFTA in the cortical area as mild (10%–25%), moderate (26%–50%), or severe (>50%) (13).

In this cohort, normal FT4 levels ranged from 12 to 22 pmol/L, and thyrotropin (TSH) from 0.27 to 4.20 μIU/mL. Thyroid function status was defined using these cut-off values, as follows: (i) overt hypothyroidism was defined as elevated TSH levels in combination with reduced FT4 levels; (ii) subclinical hypothyroidism was defined as elevated serum TSH levels in combination with normal FT4 levels; (iii) euthyroidism was defined as TSH levels within the specific reference range; (iv) overt hyperthyroidism was defined as decreased TSH levels with elevated FT4 levels; and (v) subclinical hyperthyroidism was defined as decreased TSH levels without elevated FT4 levels.

Renin-angiotensin-aldosterone system (RAAS) blocker use was defined as treatment with angiotensin-converting enzyme inhibitors, angiotensin receptor blockers, spironolactone, or any combination. Immunosuppressive treatments, including glucocorticoids and other immunosuppressants, were recorded based on the intention to treat.

### Follow-up

Serum creatinine levels were examined at least every 3 months. The primary composite endpoint included a ≥50% reduction in the eGFR, RRT initiation, or death from any cause. The eGFR and time to initiation of RRT or death were retrospectively obtained from electronic medical records.

### Statistical analysis

Continuous variables were described as mean with standard deviation (SD) and median with interquartile ranges (IQR) according to normal and non-normal distributions, respectively. Categorical variables were expressed as frequencies and constituent ratios. Depending on the distribution type of values, continuous variables were compared between groups using the Student’s *t*−test or Mann-Whitney *U*-test; categorical variables were compared using the chi-square test or the Fisher exact test. Kaplan-Meier curves were used to assess event-free survival, and the log-rank test was used to evaluate differences between groups. Univariate and multivariate Cox regression models were used to assess the association of the FT3/FT4 ratio with the composite endpoint, and crude and adjusted hazard ratios (HRs) with 95% confidence intervals (CIs) were reported. All variables in the univariate analysis were entered into the forward conditional Cox regression model for the multivariate analysis. Statistical analyses were performed using SPSS Statistics (version 25.0; IBM, Armonk, NY, USA) and GraphPad Prism 8.00 (GraphPad Software, San Diego, CA, USA). *P*-values less than 0.05 were considered statistically significant.

## Results

### Baseline characteristics

Between March 2012 and December 2014, 227 patients underwent renal biopsy at our centre and had pathological findings of glomerular crescents. After excluding 65 patients (**Figure 1**), 162 patients were included in this study. **Supplementary Table 1** summarizes the leading etiologies of background glomerular diseases based on renal biopsy and clinical information. At baseline, overt hypothyroidism, subclinical hypothyroidism, euthyroidism, subclinical hyperthyroidism and overt hyperthyroidism were present in 8, 27, 126, 1 and 0 patients, respectively. Over a median follow-up period of 47.9 (IQR 16.1–103.9) months, the primary outcome was observed in 33 patients (20.4%), including 18 patients with ≥50% reduction in eGFR, 13 patients who underwent RRT, 1 patient who died of heart failure, and 1 patient with anti-neutrophil cytoplasmic antibody-associated vasculitis that died of intracerebral haemorrhage. The distribution of the FT3/FT4 ratio is presented in **Supplementary Figure 1**, and the FT3/FT4 ratio in patients free of the primary composite endpoint was significantly higher than that in patients with the primary composite endpoint (0.269 [0.227–0.294] vs 0.247 [0.207–0.278], *P*=0.036). The incidence of the events composing the composite endpoint based on the FT3/FT4 ratio, separated by tertiles, is shown in **Supplementary Figure 2**. The incidence of events composing the primary composite endpoint was significantly lower among patients with a high FT3/FT4 ratio than among those with intermediate or low FT3/FT4 ratios (both *P* < 0.01); it was not significantly different between patients with intermediate and low FT3/FT4 ratios (*P* = 0.828). Therefore, patients were divided into high (>0.284) and low (≤0.284) FT3/FT4 ratio groups according to the first tertile.

**Table 1** summarises the baseline characteristics of the patients based on the FT3/FT4 ratio. Compared with the high FT3/FT4 ratio group, the low FT3/FT4 ratio group was older; had significantly higher urinary albumin-to-creatinine ratio (ACR), cardiac troponin T (cTnT), N-terminal brain natriuretic peptide precursor (NTproBNP), and percentage of total crescents in non-globally sclerotic glomeruli (%Cres); had higher prevalences of hypertension, moderate to severe renal tubulopathy, and crescentic nephritis; and had a higher proportion of patients receiving glucocorticoids and immunosuppressants. The low FT3/FT4 ratio group had lower haemoglobin and eGFR than the high FT3/FT4 ratio group. With regard to thyroid function tests, the low FT3/FT4 ratio group had higher FT4 levels and lower FT3 levels than the high FT3/FT4 ratio group (both *P* < 0.01); however, there were no significant differences in the TSH level or the prevalence of overt or subclinical hypothyroidism between the two groups. There were also no significant differences in sex, 24-hour urine protein, albumin, haemoglobin A1C (HbA1c), or the proportion of RAAS blocker application between the low and high FT3/FT4 ratio groups.

**Table 1:** Characteristics of patients classified according to the FT3/FT4 ratio.

Characteristics^*^	High FT3/FT4 ratio (n = 54)	Low FT3/FT4 ratio (n = 108)		*P* value
Age (years), median (IQR)	35.0 (26.8-48.0)	44.5 (31.3-57.0)	0.010			
Male, n (%)	28 (51.9)	51 (47.2)	0.578			
Urinary ACR (μg/mg), median (IQR)	863.4 (288.8-1565.5)	1140.8 (599.0-2659.7)	0.040			
24h urine protein (g), median (IQR)	1.58 (0.89-3.24)	1.96 (1.09-3.50)	0.322			
Haemoglobin (g/L), mean (SD)	130.5 (18.9)	114.4 (25.4)	<0.001			
eGFR (ml/min/1.73m^2^), median (IQR)	126.3 (85.8-142.2)	70.6 (36.5-117.6)	<0.001			
Albumin (g/L), median (IQR)	37.0 (32.0-39.0)	35.0 (29.8-38.0)	0.069			
HbA1c (%), median (IQR)	5.3 (5.1-5.6)	5.4 (5.1-5.8)	0.181			
Hypertension, n (%)	12 (22.2)	49 (45.4)	0.004			
cTnT (ng/mL), median (IQR)	0.005 (0.003-0.008)	0.007 (0.004-0.015)	0.011			
NTproBNP (pg/mL), median (IQR)	37.5 (17.9-72.9)	152.4 (37.1-445.0)	<0.001			
FT3 (pmol/L), median (IQR)	4.7 (4.1-5.0)	3.8 (2.9-4.3)	<0.001			
FT4 (pmol/L), mean (SD)	14.8 (2.4)	16.1 (2.8)	0.005			
TSH (μIU/mL), median (IQR)	2.765 (1.736-4.575)	2.615 (1.520-3.723)	0.156			
Overt and subclinical hypothyroidism, n (%)	16 (29.6)	19 (17.6)	0.079			
FT3/FT4, median (IQR)	0.30 (0.29-0.33)	0.24 (0.20-0.27)	<0.001			
%Cres (%), median (IQR)	9.8 (6.6-17.1)	14.3 (6.8-39.9)	0.014			
Moderate to severe tubulopathy, n (%)	19 (35.1)	63 (58.3)	0.005			
Crescentic nephritis, n (%)	1 (1.9)	19 (17.6)	0.004			
RAAS blocker, n (%)	22 (40.7)	35 (32.4)	0.295			
Glucocorticoids and immunosuppressants n (%)	11 (20.4)	49 (45.4)	0.002			

Continuous variables were expressed as mean (standard deviation) or median (interquartile range) and were compared using the Student’s t−test or Mann-Whitney U-test according to normal or non-normal distributions. Categorical variables are expressed as count (percentage) and compared using Pearson’s chi-square test. *Six patients were not measured for urinary ACR; 7 patients were not measured for haemoglobin; 6 patients were not measured for eGFR; 4 patients were not measured for albumin, 8 patients were not measured for HbA1c; 5 patients were not measured for cTnT; 6 patients were not measured for NTproBNP. FT3, free triiodothyronine; FT4, free thyroxine; ACR, albumin-to-creatinine ratio; eGFR, estimated glomerular filtration rate; HbA1c, haemoglobin A1C; cTnT, cardiac troponin T; NT-proBNP, N-terminal brain natriuretic peptide precursor; TSH, thyrotropin; %Cres, percentage of total crescents in non-globally sclerotic glomeruli; RAAS, renin-angiotensin-aldosterone system.

### Primary composite endpoint in high and low FT3/FT4 ratio groups

**Figure 2:**
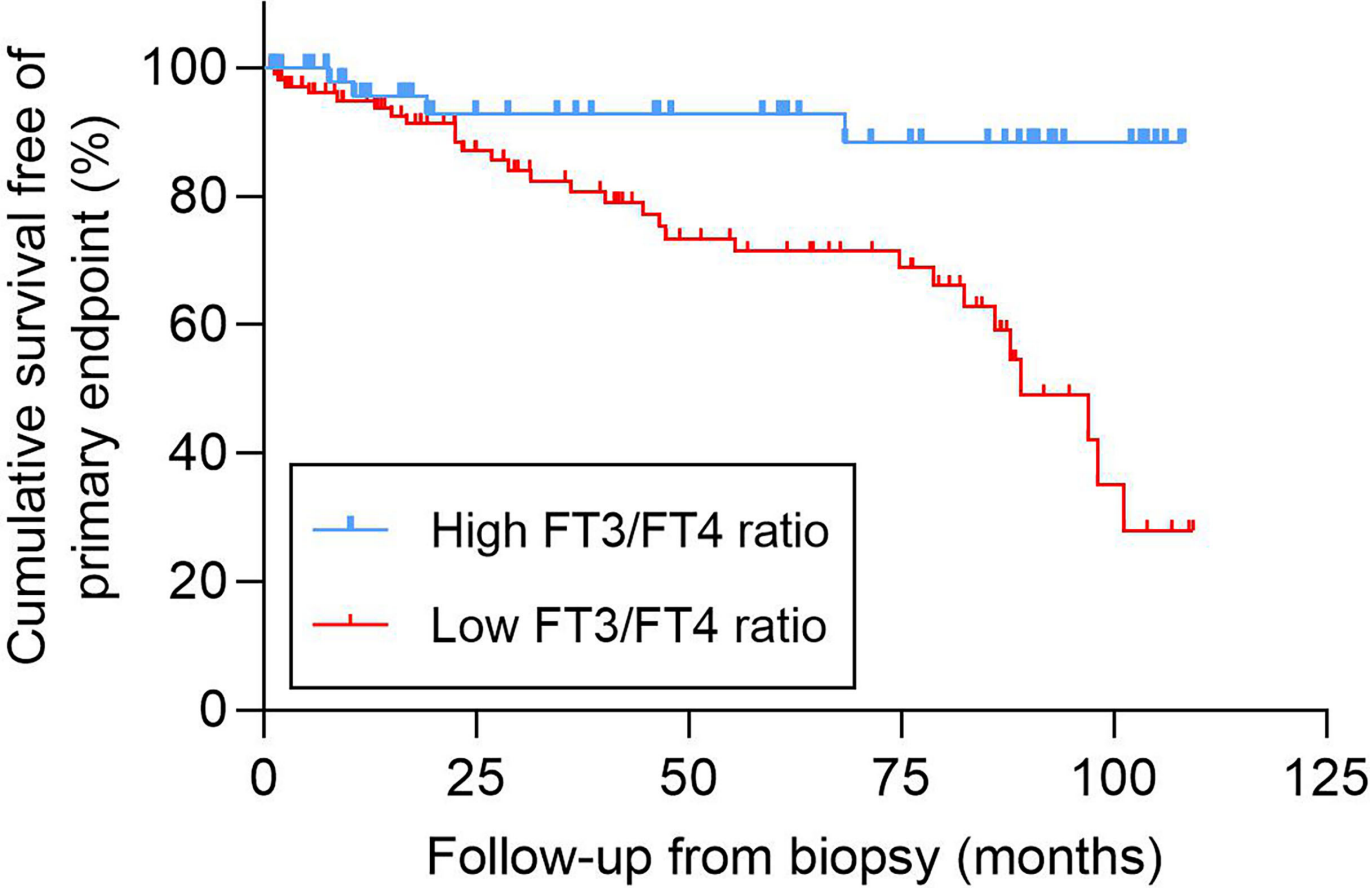
Kaplan-Meier analysis of the cumulative event-free survival according to the FT3/FT4 ratio. FT3, free triiodothyronine; FT4, free thyroxine.

The mean event-free survival was higher in the high FT3/FT4 ratio group than in the low FT3/FT4 ratio group (mean survival 99.4 months [95% CI 91.3–107.6] versus 78.3 months [95% CI 69.8–86.8], *P* = 0.001) (**Figure 2**). In the univariate Cox regression analysis, the FT3/FT4 ratio was associated with the primary composite endpoint (per 0.1-unit increase; HR = 0.549 [95% CI 0.326–0.922], *P* = 0.024). The HR of the primary endpoint increased significantly with lower haemoglobin, eGFR, and FT3; higher cTnT, NTproBNP (Ig), and %Cres; hypertension; and moderate to severe tubulopathy (all *P* < 0.01) (**Table 2**). With regard to therapy, the HR of the primary endpoint was significantly lower in patients treated with RAAS blocker (*P* < 0.05, **Table 2**). Compared to the high FT3/FT4 ratio group, the low FT3/FT4 ratio group had a higher risk of the primary composite endpoint (HR = 4.896 [95% CI 1.709–14.024], *P* = 0.003, **Table 2**). All variables in the univariate analysis were included in the forward multivariate Cox proportional hazard model. A low FT3/FT4 ratio was independently associated with the primary composite endpoint (HR = 3.079 [95% CI 1.022–9.276], *P* = 0.046, **Table 2**). In addition, hypertension was an independent risk factor for the primary outcome (HR = 3.627 [95% CI, 1.655–7.950], *P* = 0.001), whereas an elevated eGFR-EPI was an independent protective factor (per 10-unit increase; HR = 0.788 [95% CI, 0.709–0.876], *P* < 0.001) (**Table 2**). We also assessed the association of a low FT3/FT4 ratio with the composite endpoint in three multivariate Cox regression models. Model I adjusted for all variables with P < 0.1 in the univariate Cox regression analysis plus sex. Model II included all of the variables in Model I except cTnT and moderate to severe renal tubulopathy, which closely related to NTproBNP and eGFR, respectively. Model III included all of the variables from Model I except NTproBNP and moderate to severe renal tubulopathy, which closely related to cTnT and eGFR, respectively. Compared to the high FT3/FT4 ratio group, the low FT3/FT4 ratio group had an increased risk of the composite endpoint in all three models (P < 0.05) (**Table 3**).

**Table 2:** Associations between clinical, laboratory and pathological parameters and primary combined endpoint.

Variables	Univariable analysis		Multivariable analysis	
	HR (95% CI)	*P* value	HR (95% CI)	*P* value
Age	1.021 (0.997-1.045)	0.085		
Male	1.235 (0.622-2.451)	0.547		
Urinary ACR	1.110 (1.000-1.233)	0.051		
24h urine protein	1.003 (0.980-1.090)	0.226		
Haemoglobin	0.797 (0.689-0.922)	0.002		
eGFR	0.762 (0.690-0.841)	<0.001	0.788 (0.709-0.876)	<0.001
Albumin	0.966 (0.920-1.015)	0.175		
HbA1c	1.153 (0.615-2.163)	0.658		
Hypertension	5.489 (2.548-11.825)	<0.001	3.627 (1.655-7.950)	0.001
cTnT	2.238 (1.395-3.593)	<0.001		
NTproBNP (Ig)	2.595 (1.718-3.920)	<0.001		
Low FT3/FT4 Ratio	4.896 (1.709-14.024)	0.003	3.079 (1.022-9.276)	0.046
FT3	0.651 (0.486-0.872)	0.004		
FT4	0.916 (0.803-1.045)	0.191		
Overt and subclinical hypothyroidism	0.904 (0.372-2.199)	0.824		
TSH	1.021 (0.899-1.159)	0.752		
%Cres	1.207 (1.074-1.356)	0.002		
Moderate to severe tubulopathy	6.807 (2.787-16.622)	<0.001		
Crescentic nephritis	1.808 (0.783-4.176)	0.165		
RAAS blocker	0.366 (0.151-0.889)	0.026		
Glucocorticoids and immunosuppressants	1.922 (0.971-3.806)	0.061		

Urinary ACR is reported as a per 1000-unit increase; haemoglobin is reported as a per 10-unit increase; eGFR is reported as a per 10-unit increase; cTnT is reported as a per 0.1-unit increase; %Cres is reported as a per 10-unit increase. ACR, albumin-to-creatinine ratio; eGFR, estimated glomerular filtration rate; HbA1c, haemoglobin A1C; cTnT, cardiac troponin T; NT-proBNP, N-terminal brain natriuretic peptide precursor; FT3, free triiodothyronine; FT4, free thyroxine; TSH, thyrotropin; %Cres, percentage of total crescents in non-globally sclerotic glomeruli; RAAS, renin-angiotensin-aldosterone system.

**Table 3:** Multivariate Cox regression models for the primary composite outcome.

Models	HR (95%CI)	*P* value
Model I	4.635 (1.082-19.855)	0.039
Model II	4.433 (1.103-17.810)	0.036
Model III	4.660 (1.151-18.867)	0.031

The high FT3/FT4 ratio group was used as the control. Model I: Adjusted for age, sex, urinary ACR, haemoglobin, eGFR, hypertension, cTnT, NTproBNP(Ig), %Cres, moderate to severe renal tubulopathy, RAAS blockers, glucocorticoids and immunosuppressant use; Model II: Adjusted for age, sex, urinary ACR, haemoglobin, eGFR, hypertension, NTproBNP(Ig), %Cres, RAAS blocker, glucocorticoids and immunosuppressant use; Model III: Adjusted for age, sex, urinary ACR, haemoglobin, eGFR, hypertension, cTnT, %Cres, RAAS blocker, Glucocorticoids and immunosuppressant use.

## Discussion

In this observational retrospective cohort study of patients with glomerular crescents, a low baseline FT3/FT4 ratio was associated with higher renal function exacerbation and a greater risk of mortality. This prognostic association remained after multivariate adjustment for confounding factors, such as age, concomitant disease, laboratory and pathological parameters, and treatment strategy. In addition to the FT3/FT4 ratio, the presence or absence of hypertension and the eGFR at kidney biopsy were independent risk factors for the long-term composite endpoint, suggesting that baseline hypertensive comorbidity and renal function may be valuable for assessing the prognosis of CKD patients with crescent formation.

Previous studies on thyroid function in CKD patients mainly focus on the relationship between hypothyroidism and renal function and its role in renal prognosis (14–16). Thyroid hormone replacement therapy may improve renal function in patients with CKD and subclinical or overt hypothyroidism (17, 18). However, a meta-analysis of 72,856 participants with varying thyroid function found that hypothyroidism has no relationship with a faster decline in kidney function (19). Therefore, alterations in thyroid hormone metabolism in CKD may not be fully recapitulated by hypothyroidism. This was also confirmed by findings in our study that TSH level, the most sensitive biomarker of hypothyroidism, was not associated with the primary composite endpoint in univariate Cox regression analysis. And similar TSH levels between the high and low FT3/FT4 ratio groups suggested that the altered thyroid hormone metabolism in CKD with crescents was not related to hypothyroidism but rather dependent on inappropriate T4-T3 conversion.

NTIS is highly prevalent in patients with CKD, and it has value as a predictor of CKD exacerbation (20) and poor outcomes in patients with ESRD (21). In NTIS, serum FT3 is decreased relative to serum FT4 without dysregulated TSH because of decreased T4 to T3 de-iodination caused by various factors, such as inflammation and malnutrition (22). This process occurs mainly in the skeletal muscle (23). The rate of T4 to T3 de-iodination is represented by the serum FT3/FT4 ratio (24, 25), which is positively associated with muscle mass in aged, obese, and HD individuals (24–26); a lower ratio indicates impaired physical performance. Moreover, recent evidences suggest that a low FT3/FT4 ratio may be related to unfavourable prognosis in specific clinical settings. A prospective observational cohort study in a British population found that a low FT3/FT4 ratio was associated with frailty and long-term death in older hospitalised patients (27). Another study revealed that, in patients with dilated cardiomyopathy, the FT3/FT4 ratio was an independent risk factor for 1-year mortality and was associated with deteriorative heart function (28). In the context of ACS, the FT3/FT4 ratio is inversely associated with an increased risk of adverse outcomes, including all-cause and cardiac death and major adverse cardiac and cerebrovascular events (29–31). In addition, decreased FT3 and elevated FT4 levels were independent predictors of long-term mortality risk in a retrospective cohort study of hospitalised patients with chronic NTIS (32). However, few studies have focused on the relationship between the degree of T4-T3 conversion and clinical outcomes in patients with CKD. Therefore, this study provides a novel and reasonable index for predicting worsening renal function and poor prognosis in CKD patients with crescent formation, as well as a powerful supplement to the relationship between thyroid function and CKD prognosis. In addition, serum FT3 and FT4 levels are less affected by thyroid-binding globulin and serum albumin and had good stability; no additional testing is required for this ratio.

There is still no consensus on the optimal cut-off point to define a high and low FT3/FT4 ratio. Most studies categorise patients into tertiles (24, 29), as we did in our study. In studies of coronary artery disease and elderly individuals, the average values for the high tertile of the FT3/FT4 ratio ranged between 2.92 and 3.33 [(pg/mL)/(ng/dL)]; our median also fell in this range.

Age, comorbid hypertension, haemoglobin, eGFR, cTnT, NTproBNP, %Cres, moderate-to-severe tubulopathy, and administration of glucocorticoids combined with other immunosuppressive therapies are classical prognostic factors for CKD; the results of our univariate Cox analysis also showed these factors to be related to the composite endpoint. These prognostic indexes were significantly different between the high and low FT3/FT4 ratio groups. The eGFR is usually inversely associated with the severity of tubulointerstitial injury (33), and simultaneous elevation of cTnT and NTproBNP is associated with a rapid decline in kidney function and incident CKD (34). In our multivariate Cox regression models adjusted for these classic prognostic factors, a low FT3/FT4 ratio was an independent risk factor for poor prognosis in CKD patients with crescent formation, regardless of whether cTnT, NTproBNP, or moderate to severe tubulopathy were excluded.

In conclusion, we found that a low FT3/FT4 ratio was associated with poor long-term outcomes in patients with CKD with glomerular crescents. This finding suggests that the FT3/FT4 ratio is a risk factor in these patients. However, there are some unresolved questions. The mechanism by which the metabolic variation in thyroid hormones affects the progression of CKD remains to be elucidated by further basic and clinical studies. Another question is whether patients with a low FT3/FT4 ratio would benefit from a more aggressive etiological treatment or thyroid hormone replacement. Additionally, our study had several limitations. First, there were only baseline data on the FT3/FT4 ratio, and the effect of variations in this ratio during follow-up on prognosis could not be assessed. Second, it should be noted that the participants in this study were patients with CKD with crescent formation; therefore, the conclusions of this study may not be generalizable to all CKD populations. Third, information on some potential confounders, such as established cardiovascular disease or obesity, was not documented in this study; however, we had a large number of other relevant confounding variables for adjusting multivariate models.

## Data availability statement

The raw data supporting the conclusions of this article will be made available by the authors, without undue reservation.

## Ethics statement

The studies involving human participants were reviewed and approved by Ethics Committee, Zhongshan Hospital, Fudan University. Written informed consent for participation was not required for this study in accordance with the national legislation and the institutional requirements.

## Author contributions

LZ and YW were responsible for the study design. LZ, YW, YN and WL were responsible for data collection. LZ, YL and BZ were responsible for analysis and interpretation of data. LZ, SJ, ZS, FL and HL were responsible for writing the manuscript. YF and XD were responsible for supervision or mentorship. All authors were involved in writing the paper and had final approval of the submitted and published versions.

## Funding

This study was supported by the National Natural Science Foundation of China (grant 81900699), Shanghai Municipal Key Clinical Specialty (shslczdzk02501), Shanghai Municipal Hospital Frontier Technology Project supported by Shanghai ShenKang Hospital Development Center (SHDC12018127), Shanghai "science and technology innovation plan" technical standard project (19DZ2205600) and Shanghai "science and technology innovation plan" Yangtze River Delta scientific and technological Innovation Community project (21002411500).

## Conflict of interest

The authors declare that the research was conducted in the absence of any commercial or financial relationships that could be construed as a potential conflict of interest.

## Publisher’s note

All claims expressed in this article are solely those of the authors and do not necessarily represent those of their affiliated organizations, or those of the publisher, the editors and the reviewers. Any product that may be evaluated in this article, or claim that may be made by its manufacturer, is not guaranteed or endorsed by the publisher.
