# Supplementary material for: The serum free triiodothyronine to free thyroxine ratio as a potential prognostic biomarker of chronic kidney disease in patients with glomerular crescents: A retrospective study
Source: Front Endocrinol (Lausanne). 2022 Sep 29;13:977355. doi: 10.3389/fendo.2022.977355 (PMC9556952; doi:10.3389/fendo.2022.977355)
Supplement: Supplementary file 1 [file DataSheet_1.docx]

**Supplementary Table 1. the leading etiologies of background glomerular diseases classified according to the FT3/FT4 ratio**

|  | **High FT3/FT4 ratio** | **Low FT3/FT4 ratio** |
| --- | --- | --- |
| IgA nephropathy, n (%) | 43 (79.6) | 59 (54.6) |
| AAV/Anti-GBM, n (%) | 1 (1.9) | 19 (17.6) |
| lupus nephritis, n (%) | 2 (3.7) | 9 (8.3) |
| Others, n (%) | 8 (14.8) | 21 (19.4) |

***Abbreviations:*** FT3, free triiodothyronine; FT4, free thyroxine; IgA, immunoglobulin A; anti-GBM, anti-glomerular basement membrane (GBM) antibody disease; AAV, anti-neutrophil cytoplasmic antibody (ANCA)-associated vasculitis

**
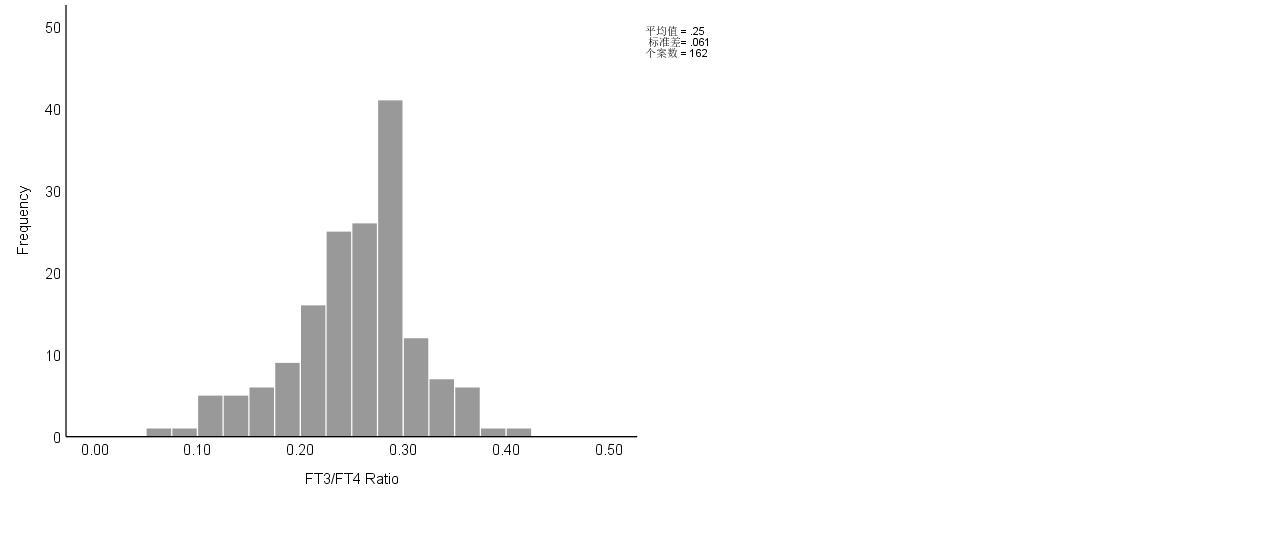
**

**Supplementary Figure 1. Distribution of FT3/FT4 ratio**

***Abbreviations:*** FT3, free triiodothyronine; FT4, free thyroxine

**Supplementary Figure 2. The incidence of events** **composing the composite endpoint based on the tertiles of FT3/FT4 ratio**

* The significance level was adjusted to 0.0125 for the three groups mutually compared using the chi-square test.

***Abbreviations:*** FT3, free triiodothyronine; FT4, free thyroxine
